# A Study on the Differences in Parental Educational Expectations and Adolescents’ Academic and Psychological Development: A Comparative Analysis of Only Children and Non-Only Children

**DOI:** 10.3390/bs15040402

**Published:** 2025-03-21

**Authors:** Guilin Xu, Yan Liu, Zhuo Tu, Xuewen Yang

**Affiliations:** 1 School of Marxism, Wuhan Textile University, Wuhan 430200, China; glxu@wtu.edu.cn; 2 School of Education, Central China Normal University, Wuhan 430200, China; tu2023110662@mails.ccnu.edu.cn; 3 Department of Curriculum and Instruction, The Education University of Hong Kong, Hong Kong, China; yxuewen@eduhk.hk

**Keywords:** educational expectations, only children, parent–child relationships, mental health, China Family Panel Studies (CFPS)

## Abstract

Based on data from the China Family Panel Studies (CFPS), this study employs structural equation modeling and multi-group path analysis to explore the mechanisms and differences in how parental educational expectations, mediated by parent–child relationships and self-educational expectations, affect the academic performance and mental health of only children and non-only children. The research reveals that parental educational expectations play a crucial role in improving adolescents’ academic performance, though their direct effect on mental health is less pronounced. Mediation effect analysis indicates that parental educational expectations have a chained mediation effect on academic performance and mental health through parent–child relationships and self-educational expectations. Multi-group path analysis reveals differences in the mediation pathways between only children and non-only children: in only-child families, the direct impact of parental educational expectations on parent–child relationships and academic performance is not significant, but the indirect effect through self-educational expectations is more prominent; in non-only-child families, parental educational expectations have a stronger direct impact on academic performance, and self-educational expectations combined with parent–child relationships exert a positive effect on mental health. This study emphasizes the crucial role of parent–child relationships and self-educational expectations in alleviating psychological stress and promoting the holistic development of adolescents. Considering the specific characteristics of different family structures, it is suggested that only-child families should focus on nurturing intrinsic motivation and alleviating the psychological stress of adolescents, while non-only-child families should enhance parent–child interaction and social support to foster the coordinated development of the academic performance and mental health of adolescents.

## 1. Introduction

The adjustment of the One-Child Policy and the relaxation of fertility policies have led to the re-emergence of multi-child families. The One-Child Policy, introduced in the late 1970s, was a population control measure that restricted most families to having only one child (Huang et al., 2019). In 2016, the policy was adjusted to allow two children, and in 2021, it was further relaxed to permit three children. These adjustments not only affect the demographic structure, labor supply, and national development, but also influence parenting styles and child development (Cai & Feng, 2021). In this context, whether changes in family structure lead to differences in parenting styles and child development outcomes across families with varying numbers of children has become a significant research topic.

A wealth of research has explored the relationship between family size and child development. Becker and Lewis (1973) introduced the Quantity–Quality Trade-off Theory, which posits that when resources are limited, an increase in family size may dilute the resources available to each child, thereby negatively impacting their education and health. In this context, the Resource Dilution Hypothesis further argues that the expansion of family size leads to the dispersion of parental resources (such as time, financial support, and emotional investment), which may result in differences between only-child and multiple-child families in terms of educational opportunities and emotional well-being (Blake, 1981). Although these theories provide valuable perspectives, they primarily focus on the distribution of material resources, neglecting the crucial role of psychological and relational dynamics in the process of adolescent development. The Family Systems Theory provides a complementary theoretical framework for understanding this research by emphasizing the interactions within family subsystems (e.g., parent–child and sibling relationships) and their collective influence on individual development (Cox & Paley, 1997). This theory suggests that changes in family size require families to adjust their relational dynamics, which may lead to differences between only-child and multiple-child families in parenting practices, resource allocation, and child development outcomes.

In China, the One-Child Policy and its subsequent relaxation have created unique conditions for studying family dynamics. Some studies find that only children may face higher mental health risks, such as higher rates of suicidal ideation and self-harming behaviors (M. Fan, 2016; Rao et al., 2024). However, other research suggests that only children have better mental health than their non-only-child peers, highlighting significant variability in the findings (Chi et al., 2020). This inconsistency in findings underscores the importance of exploring how changes in family structure impact adolescent development, particularly through the lenses of cultural background and social policy.

Parental educational expectations are widely recognized as an external source of motivation influencing both children’s academic performance and mental health. On the one hand, moderate educational expectations can stimulate children’s academic motivation and be seen as a form of challenge stress, which can enhance performance and well-being when perceived as manageable (Eccles & Wigfield, 2002); on the other hand, if these expectations surpass children’s abilities or are not supported by sufficient family resources, they may become hindrance stress, leading to increased psychological stress, such as depression and anxiety, which can hinder academic performance (Lizarte Simón et al., 2024; Xu et al., 2024). Exploring whether differences in parental educational expectations exist between only-child and non-only-child families, and whether such differences impact students’ educational development and mental health, is a key issue for both theoretical and empirical research. However, current studies on the impact of parental educational expectations on adolescents’ mental health remain limited, especially with regard to the comparison between only children and non-only children.

Based on this, this study aims to use data from the 2020 China Education Panel Survey (CEPS) to analyze the differences in academic performance and mental health between only children and non-only children in the context of contemporary China. It investigates the mechanisms through which parental educational expectations affect adolescent development via parent–child relationships and self-educational expectations. Additionally, the study provides empirical support for understanding parenting practices across family structures and their impact on adolescent development, while offering insights for optimizing family education practices and formulating tailored educational policies.

## 2. Literature Review

### 2.1. The Impact of Parental Educational Expectations and Self-Educational Expectations on Adolescent Development

Parental educational expectations refer to parents’ realistic views or assessments of their children’s future achievements, which are reflected in academic indicators such as grades and the highest level of education attained (Yamamoto & Holloway, 2010). Research indicates that the educational expectations of key individuals (like parents and peers) influence how family background factors affect an individual’s social mobility. For example, parents with optimistic educational expectations may help their children achieve better academic outcomes, surpassing outcomes predicted by family socioeconomic status alone, while parents with pessimistic educational expectations may lead their children to achieve lower academic outcomes (Jacob & Wilder, 2010). From this viewpoint, the way children progress in their learning and engage with the educational system mirrors the impact of their parents’ educational expectations. Conveying positive achievement expectations and fostering a positive academic self-concept appear to be more effective than parents directly intervening in their children’s academic outcomes, such as through homework supervision (Pinquart & Ebeling, 2020). While parental educational expectations are linked to improved academic performance in adolescents, their direct impact may be limited (X. Fan & Chen, 2001). Some studies suggest that the influence of parental expectations on adolescents’ academic performance is mediated by adolescents’ own educational expectations (T. Liu et al., 2020), highlighting the role of self-educational expectations as an important intermediary. Additionally, parental expectations may influence adolescents’ engagement in learning, serving as a potential mediating factor (Yamamoto & Holloway, 2010).

Parental educational expectations not only affect adolescents’ academic performance but also have a significant impact on their mental health (Mueller & Abrutyn, 2016). Psychological and sociological research indicates that moderate levels of parental educational expectations can enhance adolescents’ well-being. For example, Lu et al. (2021) find that positive parental expectations are significantly correlated with adolescents’ subjective well-being (SWB). However, when parental expectations surpass children’s actual abilities, they may negatively impact adolescents’ mental health. A study by Luthar et al. (2020) indicates that unrealistically high expectations can reduce adolescents’ learning motivation and increase mental stress and educational anxiety. Additionally, Man et al. (2021) find that a mismatch between parental expectations and adolescents’ abilities is a significant trigger for depressive emotions. Although existing research has highlighted the impact of parental educational expectations on adolescents’ mental health, most studies focus primarily on their direct effects on well-being, with few examining the role of self-educational expectations as a mediating variable. Moreover, empirical research based on Chinese data is relatively scarce, particularly studies that analyze the mechanisms of parental educational expectations and mental health from the perspectives of cultural background and family structure.

Based on the above, this study proposes the following research hypotheses:

**H1:**  *Self-educational expectations mediate the relationship between parental educational expectations and adolescents’ academic performance.*

**H2:**  *Self-educational expectations mediate the relationship between parental educational expectations and adolescents’ mental health.*

### 2.2. The Impact of Parental Educational Expectations and Parent–Child Relationships on Adolescent Development

Parent–child relationships are an important form of social capital between parents and children, significantly influencing adolescents’ academic performance and mental health (Coleman, 1988). Research indicates that the quality of parent–child relationships plays a significant role in adolescents’ academic achievement (Malczyk & Lawson, 2019). In families with good parent–child relationships, adolescents are more likely to receive academic support from their parents and demonstrate higher learning motivation and achievement (Shao & Kang, 2022). Within the family system, parent–child relationships serve as a key mediating variable. Parental educational expectations influence children’s development through these relationships, enhancing educational motivation and psychological adaptation via academic support and emotional care (L. Li & Liu, 2022; Wang & Roksa, 2023). This mechanism is particularly important in the context of the intense pressure within the Chinese educational system.

In other words, parent–child relationships not only influence academic performance but also play a crucial role in protecting adolescents’ mental health. Studies show that good parent–child relationships can reduce levels of depression and anxiety in adolescents and improve their subjective well-being (X. Cao & Liu, 2023; C. Li et al., 2020). Particularly in families with high educational pressure, supportive parent–child relationships can mitigate the negative effects of parental educational expectations through emotional support, enabling adolescents to better cope with the challenges posed by high expectations (Almroth et al., 2019). However, unrealistic educational expectations imposed by parents may lead to strained parent–child relationships, increasing the risk of mental health issues in adolescents. For instance, a study in Canada finds that unreasonably high expectations can exacerbate conflicts between parents and children, making adolescents more prone to self-harm behaviors (Guérin-Marion et al., 2023).

Parent–child relationships and self-educational expectations do not function in isolation; they form a synergistic effect through mutual enhancement. Good parent–child relationships can improve adolescents’ self-educational expectations by providing emotional support and boosting confidence, enabling them to have more ability to set and pursue personal academic goals (Shao & Kang, 2022). In this process, supportive parent–child relationships not only mitigate the negative effects of high parental expectations on mental health but also promote academic performance by enhancing self-educational expectations.

In summary, parent–child relationships play a crucial mediating role in the link between parental educational expectations and adolescent development, particularly in supportive family environments where their impact is more pronounced. Based on this, this study proposes the following hypotheses:

**H3:**  *Parent–child relationships mediate the relationship between parental educational expectations and adolescents’ academic performance.*

**H4:**  *Parent–child relationships mediate the relationship between parental educational expectations and adolescents’ mental health.*

**H5:**  *Parent–child relationships and self-educational expectations have a chain mediating effect on the relationship between parental educational expectations and adolescents’ academic performance.*

### 2.3. Differences Between Only Children and Non-Only Children

The Resource Dilution Hypothesis suggests that in families with multiple children, resources (such as time, emotional investment, and financial support) are diluted as the number of children increases, meaning each child receives fewer resources. In contrast, only children benefit from the full emotional and material support of their parents, giving them a significant resource advantage (Blake, 1981, 2022). For instance, in the allocation of emotional resources, mothers’ positive interactions with older children significantly decrease when a new sibling is added to the family (McHale et al., 2012). Therefore, only children typically enjoy higher-quality parent–child relationships in family education, with stronger emotional bonds with their parents and more frequent communication (Y. Liu & Jiang, 2021). Supportive parent–child relationships create a favorable learning environment for only children and enhance their sensitivity to parental educational expectations, making them more likely to actively respond to their parents’ expectations (Chiang, 2020). In other words, only children not only bear higher and more concentrated parental educational expectations but they are also more likely to internalize these expectations as personal academic goals, leading to higher self-educational expectations (Pinquart & Ebeling, 2020). However, these highly concentrated educational expectations can also impose greater psychological stress on only children, especially when the expectations exceed their abilities, potentially leading to depression and anxiety (Ma et al., 2018). Nevertheless, despite the challenges posed by resource dilution, sibling relationships can serve as a vital source of emotional support and social learning. Strong sibling bonds have been found to mitigate some of the negative effects associated with limited parental resources, fostering resilience and contributing to improved psychological well-being (Whiteman et al., 2011).

Based on the above theories and research findings, this study proposes the following hypothesis:

**H6:**  *The mediating role of parent–child relationships and self-educational expectations between parental educational expectations and adolescents’ academic performance and mental health differs between only children and non-only children.*

## 3. Method

### 3.1. Data Source and Participants

This study utilizes data from the 2020 China Family Panel Studies (CFPS), conducted by the China Social Science Survey Center at Peking University, and is supplemented with key explanatory variables from 2016 and 2018. The CFPS includes four types of questionnaires covering communities, families, adults, and children; utilizes random sampling; and covers 25 provinces, municipalities, and autonomous regions in China. This survey has strong national representativeness and reflects the overall social, economic, demographic, educational, and health conditions in China, thus holding significant scientific research value. The sample selection process in this study followed these steps: First, adolescents aged 10–15 were selected as the study subjects due to the inclusion of both self-reported and parent-reported data, with self-educational expectations reported by adolescents and academic performance and parental educational expectations reported by parents, and this age group is more suitable, with superior selection characteristics compared to younger primary school students. Second, parent questionnaires were matched using child IDs, and samples with missing key analysis variables were excluded. Ultimately, the effective sample size included in the model analysis was 1376.

### 3.2. Operational Definition of Variables

Academic Performance: Adolescents’ academic performance is measured by the average of their Chinese and math grades for the previous semester, as reported by parents in 2020. The grades are rated on a scale where 1 indicates “poor”, 2 indicates “average”, 3 indicates “good”, and 4 indicates “excellent”. This variable has been standardized, with values ranging from 1 to 4, where higher values denote better academic performance.

Mental Health: In this study, the mental health indicator is measured using the CES-D8, a simplified version of the Center for Epidemiologic Studies Depression Scale (CES-D) originally developed by Radloff (1977). Due to concerns about survey fatigue and response accuracy, the CFPS has adopted the 8-item short version (CES-D8) since 2018 to improve questionnaire completion rates. The respondents in this study were adolescents, who self-reported their psychological well-being using the CES-D8. This short version has been widely validated and successfully applied in studies on adolescent mental health across various populations (S. Liu et al., 2023). The CES-D8 scale covers the following dimensions: depressive mood (e.g., “feeling depressed”, “feeling lonely”, “finding life difficult”), life enjoyment (e.g., “feeling happy”, “enjoying life”), and sleep quality (e.g., “poor sleep quality”). Respondents answer questions based on the frequency of their emotions and behaviors in the past week, with options ranging from “rarely” (1 point) to “most of the time” (4 points). Negative items such as “feeling depressed” and “feeling lonely” are reverse-scored. The final score is calculated by averaging the total scores of the eight items, with higher scores indicating better mental health. To ensure the reliability and validity of the measurement, this study follows recommendations from related literature (D. Cao et al., 2022) and employs factor analysis and principal component analysis for robustness tests.

Parental Educational Expectations: Parental educational expectations refer to the educational attainment parents expect their children to achieve in the future. There are eight options, converted based on years of education and degree levels: 0 = no schooling required, 6 = primary school, 9 = junior high school, 12 = senior high school, 15 = junior college, 16 = bachelor’s degree, 19 = master’s degree, and 23 = doctoral degree. This single-item measure has been widely used in adolescent research (Froiland et al., 2013; Pinquart & Ebeling, 2020). Studies show that this measure has high reliability and predictive validity, with parental educational expectations in early childhood serving as a reliable predictor for subsequent educational aspirations and academic performance across various subjects in longitudinal studies (Froiland et al., 2013).

Self-Educational Expectations: Self-educational expectations refer to the educational attainment that students themselves expect to achieve in the future. This variable is quantified using the same method as parental educational expectations. It is a continuous variable, with higher values indicating higher educational expectations.

Parent–Child Relationships: The parent–child relationships are assessed from three dimensions: conflict, intimacy, and dependency (J. Liu et al., 2024; Shi et al., 2022). Conflict is measured by the frequency of intense arguments between children and their parents. The questionnaire responses are categorized into an ordinal variable and reverse-scored: no arguments in the past month is scored as 3 (low frequency), 0–4 arguments as 2 (moderate frequency), and more than 4 arguments as 1 (high frequency). Intimacy is evaluated through two indicators: the frequency of heart-to-heart conversations between children and parents (scored 1–3 based on the number of conversations in the past month), and the frequency of discussions about school matters (retaining the original five-category variable from the questionnaire). Dependency is measured by the willingness of children to confide in their parents when encountering troubles. Responses coded as “willing” are assigned a value of 1, while other responses are coded as 0. A composite parent–child relationship variable is generated by extracting common factors from these three dimensions through principal component analysis. Higher scores indicate a closer parent–child relationship.

### 3.3. Measures

The data analysis consists of three parts. Firstly, SPSS 25.0 is used for descriptive statistics and correlation analysis to reflect the overall characteristics of the number of children per family, and to examine the relationships between parental educational expectations, self-educational expectations, parent–child relationships, adolescents’ academic performance, and mental health. Secondly, Amos 24.0 is employed to establish structural equation models and to perform mediation model tests to estimate the chain mediating effects of self-educational expectations and parent–child relationships on the influence of parental educational expectations on adolescents’ academic performance and mental health. Finally, based on multi-group analysis methods, this study explores the differences in mediation model paths between only-children and non-only-children groups to reveal the similarities and differences in the mechanisms of action between these two groups.

## 4. Results

### 4.1. Common Method Bias Test

This study employed Harman’s single-factor test to assess the potential presence of common method bias. The results indicated that two factors, each with an eigenvalue greater than 1, were extracted, together accounting for 57.52% of the variance. Of these, the first factor explained 36.14% of the variance, which is below the 40% critical threshold. Thus, no significant common method bias was detected in this study (Lindell & Whitney, 2001).

### 4.2. Differences Between Only Children and Non-Only Children in Parental Educational Expectations, Parent–Child Relationships, Self-Educational Expectations, Academic Performance, and Mental Health

Table 1 presents the analysis of differences between only children and non-only children across various variables. The results reveal the following: First, only children exhibit significantly higher parental educational expectations and academic performance than non-only children (*t* = 3.68, *p* < 0.01; *t* = 2.52, *p* < 0.05). Second, no significant differences were found between the two groups concerning parent–child relationships, self-educational expectations, and mental health. Third, there are significant differences between only children and non-only children in terms of gender composition, household registration, and family income. Specifically, the gender distribution among only children is 61.1% male and 38.9% female, while the gender distribution among non-only children is 51.1% male and 48.9% female (*t* = 2.75, *p* < 0.01). Among only children, 56.5% have rural household registration, whereas 84.7% of non-only children have rural household registration (*t* = −9.872, *p* < 0.00). In terms of family income, 22.7%, 26.9%, and 50.5% of only children belong to low-, medium-, and high-income families, respectively, while for non-only children, the percentages are 28.3%, 37.8%, and 33.9%, respectively (*t* = 3.41, *p* < 0.01).

### 4.3. Correlation Between Parental Educational Expectations, Parent–Child Relationships, Self-Educational Expectations, Academic Performance, and Mental Health

A correlation analysis was performed to explore the relationships among parental educational expectations, self-educational expectations, parent–child relationships, academic performance, mental health, and other demographic variables. As indicated in Table 2, significant correlations were observed among the five primary variables—parental educational expectations, self-educational expectations, parent–child relationships, academic performance, and mental health. These findings meet the preliminary conditions required to test for mediating effects. Moreover, despite the presence of significant correlations, none of the coefficients exceed 0.8, indicating that multicollinearity is not a notable issue.

### 4.4. Chain Mediation Effect of Parent–Child Relationships and Self-Educational Expectations on the Link Between Parental Educational Expectations and Academic Performance and Mental Health

**Figure 1:**
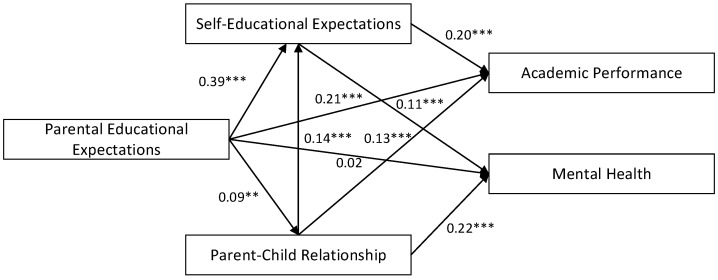
Path coefficients of the model. Note: ** *p* < 0.01, *** *p* < 0.001.

A chain mediation model was constructed using AMOS 24 to investigate the relationship between parental educational expectations, parent–child relationship, self-educational expectations, academic performance, and mental health. In this model, parental educational expectations served as the independent variable, parent–child relationship and self-educational expectations were employed as mediating variables, and academic performance and mental health were designated as the outcome variables. Demographic factors, including age, gender, household registration, and family income, were incorporated as control variables. Structural equation modeling (SEM) was applied to analyze the model, which included two outcome variables. The fit indices indicated that the model demonstrated a good fit with the data (χ^2^/df = 5.39, RMSEA = 0.056, CFI = 0.993, TLI = 0.928, SRMR = 0.014). The specific path coefficients are illustrated in Figure 1.

To test the mediation effect, the bias-corrected percentile Bootstrap method was utilized, with 5000 resampling iterations. The statistical significance of the indirect effect was determined by examining whether the confidence interval (CI) excluded zero, thereby confirming the presence of a mediation effect (Taylor et al., 2008). The results, as shown in Table 3, revealed that parental educational expectations exerted a significant positive influence on adolescents’ academic performance (β = 0.08, *p* < 0.001), while the direct effect on mental health was not statistically significant. The analysis further indicated that parental educational expectations indirectly affected both academic performance and mental health through the mediating roles of parent–child relationship and self-educational expectations.

More specifically, the indirect effect of parental educational expectations on academic performance through the parent–child relationship was statistically significant (β = 0.011, 95% CI: [0.005–0.022]), accounting for 3.7% of the total effect. Additionally, the indirect effect through self-educational expectations was also significant (β = 0.077, 95% CI: [0.056–0.102]), contributing 25.6% to the total effect. In relation to mental health, parental educational expectations exhibited a significant indirect effect via the parent–child relationship (β = 0.019, 95% CI: [0.008–0.034]), representing 21.8% of the total effect, and through self-educational expectations (β = 0.042, 95% CI: [0.018–0.067]), which accounted for 48.3% of the total effect. Furthermore, the results highlighted the existence of a chain mediation effect, wherein parental educational expectations influenced academic performance through the sequential mediation of the parent–child relationship and self-educational expectations (β = 0.002, 95% CI: [0.001–0.005]), accounting for 0.7% of the total effect. A similar chain mediation effect was observed for mental health (β = 0.001, 95% CI: [0.000–0.003]), contributing 1.1% to the total effect. These findings provide robust empirical support for hypotheses 1 through 5, validating the chain mediation model and underscoring the critical role of the parent–child relationship and self-educational expectations in mediating the influence of parental educational expectations on adolescents’ academic performance and mental health.

### 4.5. Multi-Group Analysis of the Chain Mediation Model

A multi-group path analysis was conducted to explore differences in the chain mediation model between only-child and non-only-child groups, with only-child status serving as the grouping variable. Model fit was assessed by progressively comparing models, from the least restrictive to the most restrictive. The results indicated that the fit indices for the unconstrained model, referred to as the baseline model, as well as the structural weights, structural covariances, and structural residuals models, were all within acceptable ranges. The primary objective of the study was to examine the moderating role of family structure, specifically the distinction between only-child and non-only-child families, in the pathways linking parental educational expectations, the parent–child relationship, and self-educational expectations to academic performance and mental health. To this end, the invariance of the structural weights model across the two family structures was evaluated. A chi-square difference test comparing the unconstrained and restricted models revealed no significant differences between the model with equal structural coefficients and other nested models, under the assumption that the baseline model was valid (Δχ^2^ = 15.351, ΔDF = 9, *p* > 0.05). This result suggests that the structural weights model is invariant across both groups, indicating that the overall path coefficients do not differ significantly between only-child and non-only-child groups.

Despite the invariance of the overall model, further analysis of specific path coefficients revealed notable differences between the two groups (see Table 4 and Table 5). In the non-only-child group, parental educational expectations had a significant positive influence on both the parent–child relationship and academic performance. This suggests that higher parental expectations not only enhance the quality of the parent–child relationship but also directly promote adolescents’ academic achievement. In contrast, for the only-child group, parental educational expectations did not have a significant impact on either the parent–child relationship or academic performance, indicating that in only-child families, parental expectations may not translate into stronger parent–child bonds or improved academic outcomes. Regardless of family structure, the analysis revealed that parent–child relationship and self-educational expectations consistently exerted significant positive effects on academic performance and mental health. Adolescents with stronger parent–child relationships and higher self-educational expectations demonstrated better academic performance and experienced fewer mental health issues. However, the direct effect of parental educational expectations on mental health was not statistically significant in either group, suggesting that while parental expectations may indirectly contribute to better mental health through other mediators, their direct influence is limited. The findings of this study provide partial support for Hypothesis 6, reinforcing the importance of parent–child relationships and self-educational expectations as mediators in the relationship between parental educational expectations and adolescent outcomes. At the same time, the differences observed between only-child and non-only-child families highlight the nuanced ways in which family dynamics may shape the pathways linking parental expectations to developmental outcomes.

## 5. Discussion

### 5.1. Analysis of Differences in Parental Educational Expectations, Parent–Child Relationships, Self-Educational Expectations, Academic Performance, and Mental Health

The findings indicate that parental educational expectations are significantly higher in only-child families compared to non-only-child families. This suggests that parents in only-child households place greater emphasis on their child’s academic success and future achievements. Conversely, parents in non-only-child families may need to allocate educational resources across multiple children, resulting in slightly lower overall expectations. This observation aligns with the higher economic status typically associated with only-child families, as reflected by data showing that family income in these households is significantly greater than in non-only-child households. The findings support the Resource Dilution Theory, which posits that in larger families, limited resources—such as financial support, parental attention, and emotional investment—are distributed among multiple children, potentially diminishing the resources available to each individual.

Regarding parent–child relationships, the study found no significant differences between only-child and non-only-child families. This contrasts with the predictions of Resource Dilution Theory concerning relational dynamics and suggests that, irrespective of family size, parents make concerted efforts to maintain close and supportive relationships with their children. This is consistent with contemporary trends in family education, which emphasize the importance of fostering strong parent–child bonds (Yaffe et al., 2024). While self-educational expectations were marginally higher among adolescents from only-child families, the difference was not statistically significant. This implies that adolescents’ self-educational expectations are influenced more by external factors, such as personality traits and school environments, rather than solely by family structure (Basharpoor et al., 2022). A noteworthy finding is the significantly higher academic performance observed among only children compared to their non-only-child counterparts, supporting the existing literature (M. Li et al., 2021). This advantage may be attributed to the greater educational focus and resource investment directed toward only children. Additionally, the higher economic standing and stronger educational support systems in only-child households likely contribute to this outcome. In terms of mental health, no significant differences were detected between only children and non-only children. This suggests that only children do not exhibit discernible disadvantages in mental health relative to adolescents with siblings. These results align with the research of Rao et al. (2024), indicating that family size exerts a limited direct influence on mental health, with individual and environmental factors playing a more critical role.

### 5.2. The Relationship Between Parental Educational Expectations, Parent–Child Relationships, Self-Educational Expectations, Academic Performance, and Mental Health

The analysis demonstrates that parental educational expectations significantly influence students’ academic performance, with this effect being further reinforced by the mediating roles of the parent–child relationship and self-educational expectations. Parental expectations exert a direct and notable positive impact on academic performance, while also enhancing outcomes indirectly through pathways involving parent–child relationships and self-educational expectations. These findings suggest that high parental expectations are not merely a reflection of available educational resources but actively contribute to academic performance by fostering a supportive family environment and strengthening adolescents’ self-motivation. This highlights the systemic nature of educational expectations, which extend beyond parental beliefs to shape student development through family interactions and individual cognition (Wigfield & Eccles, 2000; Yamamoto & Holloway, 2010).

In terms of mental health, although the direct effect of parental educational expectations is not statistically significant, their indirect influence through parent–child relationships and self-educational expectations is pronounced. This study reveals that parental expectations contribute to improved mental health primarily by strengthening parent–child relationships and, to a greater extent, by fostering higher self-educational expectations. This underscores the role of family dynamics and self-expectations in mitigating the psychological pressure associated with parental demands, thereby promoting adolescent well-being (Almroth et al., 2019). Importantly, the mediating effect of self-educational expectations on mental health surpasses that of the parent–child relationship, indicating that autonomy, competence, and belongingness are critical to mental well-being. When students internalize academic goals as self-directed rather than externally imposed, they experience enhanced psychological satisfaction and resilience. This reduces the burden of parental expectations and allows students to perceive academic pursuits as personally fulfilling (T. Li & Wang, 2024). Research further supports the notion that balancing parental expectations with adolescent autonomy alleviates anxiety, fosters confidence, and enhances mental health (Zhang, 2024).

In conclusion, the effect of parental educational expectations on academic performance is primarily direct (accounting for 69.8% of the total effect), while their influence on mental health operates predominantly through the mediating pathways of the parent–child relationship and self-educational expectations (accounting for 71.3% of the total effect). These findings emphasize both the direct contribution of parental expectations to academic success and the complex mechanisms through which they shape mental health outcomes.

### 5.3. Differences in the Relationship Between Parental Educational Expectations, Parent–Child Relationships, Self-Educational Expectations, Academic Performance, and Mental Health Among Only Children and Non-Only Children

The group analysis reveals no significant differences in overall path coefficients between only children and non-only children, indicating that the mechanisms by which parental educational expectations influence academic performance and mental health through parent–child relationships and self-educational expectations operate similarly across the two groups. However, a closer examination of the path coefficients highlights notable distinctions in specific pathways. In terms of academic performance, parental educational expectations do not exert a significant direct effect in only-child families. Drawing from Expectation-Stress Theory, this outcome suggests that excessive parental expectations can induce considerable pressure, potentially hindering performance—particularly when children perceive the expectations as unattainable (Lazarus, 1984). This may account for the lack of a direct association between elevated parental expectations and academic success in only-child households.

Further analysis underscores the greater reliance of only children on self-educational expectations to drive academic achievement. Data indicate that 41.03% of the influence of parental educational expectations on academic performance in only-child families is mediated through self-educational expectations, compared to just 3.85% mediated by the parent–child relationship. The concentrated focus of parents on their only child fosters the internalization of these expectations, strengthening intrinsic motivation and personal goal-setting. This implies that academic success is more likely when students possess autonomy and self-driven goals (Eccles & Wigfield, 2002; Pinquart & Ebeling, 2020). Consequently, the primary driver of academic performance in only-child families is self-educational expectation, while the direct influence of parental expectations remains limited. In contrast, non-only-child families present a different pattern. Despite lower overall parental educational expectations, their direct impact on academic performance is more substantial, accounting for 71.99% of the total effect—significantly higher than in only-child families. This aligns with Balanced Expectation Theory, which posits that moderate levels of expectation effectively enhance motivation, whereas excessively high or low expectations may undermine performance (Wigfield & Eccles, 2000).

Regarding mental health, the mechanisms by which parental educational expectations exert influence are consistent across both groups, with parent–child relationships and self-educational expectations serving as significant mediators. In only-child families, the indirect effect of parental educational expectations on mental health is particularly pronounced, with self-educational expectations playing a dominant role in alleviating psychological pressure and enhancing well-being. Parent–child relationships also contribute significantly by providing essential emotional support, thereby mitigating the unique pressures experienced by only children. In non-only-child families, while the mediation effect remains substantial, it is relatively less pronounced compared to only-child families. This suggests that non-only children benefit from a combination of strong family bonds and self-motivation in maintaining mental health, with self-educational expectations still serving as a key influencing factor. Interestingly, in both groups, the direct effect of parental educational expectations on mental health is not statistically significant. However, the negative effect observed in only-child families suggests that concentrated parental expectations may impose a psychological burden on only children, potentially resulting in adverse mental health outcomes.

## 6. Conclusions

This study expands on existing research by investigating the pathways through which parental educational expectations influence adolescents’ academic performance and mental health, focusing on the mediating roles of self-educational expectations and parent–child relationships. Previous research highlights the critical influence of parental expectations on adolescent development, particularly within the framework of intergenerational transmission. These studies also emphasize differences between only-child and non-only-child families, noting that only children often experience greater pressure due to concentrated parental expectations, while non-only children benefit from sibling support, fostering stronger emotional regulation. Despite these insights, comparative studies examining the influence of parental expectations across different family structures remain scarce. Through cross-group comparative analysis, this study reveals the mechanisms by which parental expectations shape academic performance and mental health in families of varying sizes, underscoring the chain mediation effects of self-educational expectations and parent–child relationships. These findings provide novel perspectives on the distinct and overlapping dynamics in only-child and non-only-child families, establishing a theoretical framework to address the implications of evolving family planning policies.

In only-child families, heightened parental expectations may impose significant psychological pressure. However, self-educational expectations and parent–child relationships play vital roles in mitigating this pressure, promoting mental well-being. This underscores the necessity for parents to balance high expectations with a recognition of their child’s need for autonomy and emotional support. By fostering intrinsic motivation and maintaining healthy parent–child relationships, only-child families can better support adolescents in navigating academic and mental health challenges. In contrast, non-only-child families experience more pronounced effects of resource dilution, wherein parental time, financial resources, and emotional support must be distributed among multiple children, potentially limiting individual academic support. To counteract these challenges, policies aimed at providing additional resources and support for multi-child families are essential. This study further illustrates the interplay between parental expectations, self-educational expectations, and parent–child relationships, highlighting their collective importance in fostering the academic and psychological well-being of non-only children.

As policies surrounding two-child and three-child families continue to evolve, understanding how to equitably distribute parental expectations and foster emotional connections with multiple children is crucial for promoting adolescent success and well-being. This study offers a practical framework for addressing the unique challenges faced by multi-child families, guiding parents in fostering positive academic and mental health outcomes without imposing excessive pressure. The findings have significant implications for educational policy and family support initiatives. Policymakers are encouraged to develop targeted interventions that promote the equitable distribution of parental expectations and enhance supportive family dynamics. In the context of shifting family structures, prioritizing fair resource allocation and emotional engagement can optimize developmental outcomes for adolescents across diverse family environments.

## 7. Limitations and Suggestions for Future Studies

This study’s reliance on cross-sectional data limits its ability to draw definitive causal conclusions. Although the mediation model offers a robust theoretical framework for understanding how parental educational expectations, self-educational expectations, and parent–child relationships influence adolescents’ academic performance and mental health, the static nature of cross-sectional data restricts the capacity to capture dynamic interactions between these variables over time. To address this, future research should adopt longitudinal designs to observe changes in parental expectations, mediating factors, and adolescent outcomes across different developmental stages. A longitudinal approach would provide greater clarity on the causal pathways and temporal evolution of these relationships, thereby strengthening the empirical foundation of the chain mediation model.

Furthermore, this study primarily focuses on self-educational expectations and parent–child relationships as mediators, potentially neglecting other important variables that may play a role in the relationship between parental expectations and adolescent development. Mediators such as peer relationships, teacher–student interactions, and school environments may also exert significant influence on adolescents’ academic performance and mental health. Future studies should broaden the investigation to include factors like social support, emotional regulation, and extracurricular involvement. Additionally, this study did not incorporate parental demographic factors such as age, educational background, and socioeconomic status, as they were not within the scope of our research focus. However, these factors may influence parental expectations and involvement in children’s education, thereby affecting adolescent academic and psychological outcomes. Future research should consider integrating these parental characteristics to provide a more comprehensive perspective on family influences. By expanding the scope of mediating variables, future research can provide a more comprehensive and nuanced understanding of the mechanisms through which parental expectations affect adolescent development, contributing to more effective strategies for fostering academic success and promoting mental health.

## Figures and Tables

**Table 1:** Differences between two family types in parental educational expectations, parent–child relationships, self-educational expectations, academic performance, and mental health (x ± s).

Variable	Only Children (216)	Non-Only Children (1160)	*t*
Parental Educational Expectations (PEE)	19.19 ± 2.24	18.56 ± 2.32	3.68 **
Parent–Child Relationship (PCR)	3.61 ± 42.18	−0.67 ± 044.76	1.30
Self-Educational Expectations (SEE)	18.15 ± 2.83	17.82 ± 2.83	1.54
Mental Health (MH)	3.55 ± 0.48	3.54 ± 0.49	0.21
Academic Performance (AP)	2.84 ± 0.84	2.68 ± 0.90	2.52 *
Age	12.09 ± 1.79	12.11 ± 1.76	−0.20
Gender (Gen)	0.61 ± 0.49	0.51 ± 0.50	2.75 **
Household Registration (HR)	0.56 ± 0.50	0.85 ± 0.36	−7.95 ***
Family Income (FI)	3.92 ± 1.62	3.49 ± 1.58	3.41 **

Note: * *p* < 0.05, ** *p* < 0.01, *** *p* < 0.001.

**Table 2:** Correlations between parental education level, parental educational expectations, self-educational expectations, academic performance, and demographic variables (r).

	x ± s	1	2	3	4	5	6	7	8
1 PEE	15.83 ± 2.51	1							
2 PCR	0 ± 44.38	0.09 **	1						
3 SEE	15.11 ± 3.05	0.41 **	0.18 **	1					
4 AP	2.70 ± 0.90	0.30 **	0.19 **	0.30 **	1				
5 MH	3.54 ± 0.49	0.09 **	0.24 **	0.16 **	0.14 **	1			
6 Age	12.11 ± 1.77	−0.00	−0.21 **	−0.07 *	−0.16 **	−0.08 **	1		
7 Gen	0.53 ± 0.50	0.00	−0.01 *	−0.06 *	−0.10 **	0.03	0.00	1	
8 HR	0.80 ± 0.40	−0.11 **	−0.03	−0.12 **	−0.06 *	−0.03	0.02	−0.01	1
9 FI	3.77 ± 1.65	0.12 **	0.03	0.14 **	0.11 **	−0.00	−0.06 *	0.01	−0.24 **

Note: * *p* < 0.05, ** *p* < 0.01.

**Table 3:** Mediation effect of self-educational expectations and parent–child relationship between parental educational expectations and academic performance/mental health.

Dependent Variable	Path	Standardized Path Effect	Effect Size/%	95% CI	
				Lower	Upper
AP	PEE → PCR → AP	0.011	3.7	0.005	0.022
	PEE → SEE → AP	0.077	25.6	0.056	0.102
	PEE → PCR → SEE → AP	0.002	0.7	0.001	0.005
	Total Indirect Effect	0.091	30.2	0.067	0.117
	Direct Effect	0.210	69.8	0.158	0.264
	Total Effect	0.301	100	0.254	0.349
MH	PEE → PCR → MH	0.019	21.8	0.008	0.034
	PEE → SEE → MH	0.042	48.3	0.018	0.067
	PEE → PCR → SEE → MH	0.001	1.1	0.000	0.003
	Total Indirect Effect	0.062	71.3	0.029	0.143
	Direct Effect	0.024	27.6	−0.036	0.086
	Total Effect	0.087	100	0.029	0.143

Note: For abbreviations, refer to Table 1.

**Table 4:** Comparison of coefficients in the chain mediation model.

Path	β	
	Only Child	Non-Only Child
PEE → PCR	0.058	0.087 **
PEE → SEE	0.345 ***	0.401 ***
PEE → AP	0.128	0.221 ***
PEE → MH	−0.067	0.041
PCR → AP	0.162 *	0.130 ***
PCR → MH	0.355 ***	0.199 ***
SEE → AP	0.278 ***	0.180 ***
SEE → MH	0.149 *	0.102 **
PCR → SEE	0.006	0.165 ***

Note: * *p* < 0.05, ** *p* < 0.01, *** *p* < 0.001. For abbreviations, refer to Table 1.

**Table 5:** Mediation effect of self-educational expectations and parent–child relationships between parental educational expectations and academic performance/mental health in only-child and non-only-child groups.

		Only Child				Non-Only Child			
				95% CI				95% CI	
DV	Path	Std. Effect	Effect Size/%	Lower	Upper	Std. Effect	Effect Size/%	Lower	Upper
AP	PEE → PCR → AP	0.009	3.85	−0.007	0.037	0.011	3.58	0.004	0.023
	PEE → SEE → AP	0.096	41.03	0.048	0.155	0.072	23.45	0.049	0.099
	PEE → PCR → SEE → AP	0.000	0	−0.001	0.004	0.003	0.10	0.001	0.005
	Total Ind. Eff.	0.105	6.41	0.020	0.064	0.086	28.01	0.023	0.045
	Dir. Eff.	0.128	54.70	0.002	0.093	0.221	71.99	0.063	0.109
	Tot. Eff.	0.234	100	0.041	0.131	0.307	100	0.098	0.141
MH	PEE → PCR → MH	0.020	400	−0.019	0.069	0.017	16.83	0.006	0.032
	PEE → SEE → MH	0.052	1040	−0.007	0.134	0.041	40.59	0.015	0.067
	PEE → PCR → SEE → MH	0.000	0	−0.002	0.005	0.003	2.97	0.001	0.005
	Total Ind. Eff.	0.072	1440	−0.001	0.035	0.060	59.41	0.006	0.020
	Dir. Eff.	−0.067	−1340	−0.043	0.017	0.041	40.59	−0.005	0.022
	Tot. Eff.	0.005	100	−0.028	0.030	0.101	100	0.009	0.034

Note: For abbreviations, refer to Table 1.

## Data Availability

The data that support the findings of this study are available from the corresponding author upon reasonable request.
